# Surgical Resection Is Still Better Than Endoscopic Resection for Patients With 2-5 cm Gastric Gastrointestinal Stromal Tumours: A Propensity Score Matching Analysis

**DOI:** 10.3389/fonc.2021.737885

**Published:** 2021-09-15

**Authors:** Hao Wu, Han Li, Qinfeng Xu, Liang Shang, Ronghua Zhang, Chen Li, Mengdi Fu, Wandi Xu, Jianfeng Chen, Jin Liu, Leping Li

**Affiliations:** ^1^Department of Gastroenterological Surgery, Shandong Provincial Hospital, Cheeloo College of Medicine, Shandong University, Jinan, China; ^2^Department of General Surgery, The First Affiliated Hospital of Shandong First Medical University, Jinan, China; ^3^Department of Clinical Medicine, Cheeloo College of Medicine, Shandong University, Jinan, China; ^4^Medical Science and Technology Innovation Center, Shandong First Medical University & Shandong Academy of Medical Sciences, Jinan, China; ^5^Department of Gastroenterological Surgery, Shandong Provincial Hospital Affiliated to Shandong First Medical University, Jinan, China; ^6^Department of Digestive Tumor Translational Medicine, Engineering Laboratory of Shandong Province, Shandong Provincial Hospital, Jinan, China; ^7^Department of Gastroenterological Surgery, Peking University People’s Hospital, Beijing, China; ^8^Department of Gastroenterology, Shandong Provincial Hospital, Cheeloo College of Medicine, Shandong University, Jinan, China; ^9^Research Center for Experimental Nuclear Medicine, School of Basic Medical Sciences, Shandong University, Jinan, China

**Keywords:** gastrointestinal stromal tumours, surgical, endoscopic, propensity score matching, gastric tumours

## Abstract

**Background:**

The management of 2-5 cm gastric gastrointestinal stromal tumours (GISTs) is still debated between surgeons and endoscopists. We aimed to investigate short-term and long-term outcomes between surgical resection (SR) and endoscopic resection (ER).

**Methods:**

This study included 67 and 215 patients between 2010 and 2020 who underwent ER and SR, respectively. After propensity score matching, the clinical outcomes were compared. Individual patient information that requires special instructions is also summarized.

**Results:**

After matching, the operation time (P=0.005) and postoperative hospital stay (P=0.005) were significantly longer in the SR group than in the ER group. However, there were no significant differences in blood loss (P=0.741), resection margin (P=1.000) or time to liquid diet (P=0.055). Statistical differences were also seen in en bloc resection (P<0.001) and adverse events (P=0.027). The recurrence rate did not differ significantly between the two techniques, and the mitotic index and ulceration were identified as independent prognostic factors of progression-free survival.

**Conclusions:**

ER might be comparable to SR for the treatment of 2-3 cm gastric GISTs. SR is still considered the standard treatment for 3-5 cm gastric GISTs, while the intraoperative and postoperative information of ER should be recorded in detail and closely evaluated. Surgical resection is recommended if the tumour has a high mitotic index or mucosal ulceration.

## Introduction

As one of the most common mesenchymal neoplasms, gastrointestinal stromal tumours (GISTs) have attracted increasing attention in recent years (1–4). GISTs can be found anywhere of the gastrointestinal (GI) tract, but most appear in the stomach (5). Although the majority of gastric GISTs are indolent, all GISTs are believed to have malignant potential (6 **).**

According to the latest guidelines of the National Comprehensive Cancer Network (NCCN) (7) and European Society for Medical Oncology (ESMO) (8), gastric GISTs smaller than 2 cm without high-risk features (irregular borders, cystic spaces, ulceration, echogenic foci, heterogeneity) should receive periodic surveillance, while surgical resection (SR) is the recommended treatment for primary, localized gastric GISTs larger than 2 cm.

In recent years, endoscopic resection (ER) has gradually been used to remove small GISTSs (9). However, there is no guarantee of en bloc resection, and the potential risk of recurrence is an issue that doctors continue to pay attention to (10, 11). Moreover, many studies have compared the clinical outcomes of ER with those of SR in the treatment of gastric GISTs smaller than 2 cm, and there are currently no high-level studies supporting a clear best choice for 2-5 cm GISTs.

We aimed to compare short-term and long-term outcomes between SR and ER for 2-5 cm gastric GISTs and provide a potential reference for the standardization of the treatment.

To ensure suitable randomization in the evaluation of short- and long-term outcomes in GIST patients who underwent SR or ER, we applied propensity score matching to equalize the baseline clinicopathological characteristics.

## Patients and Methods

This retrospective cohort study was performed based on a prospectively collected database of GISTs at Shandong Provincial Hospital (SPH). All relevant procedures were approved by the Institutional Review Board (IRB). This study was designed in compliance with the Helsinki Declaration and approved by the Ethics Committee of Shandong Provincial Hospital, China (SWYX: No. 2021-035). The Reporting and Guidelines in Propensity Score Analysis were also followed (12).

### Patients

**Figure 1:**
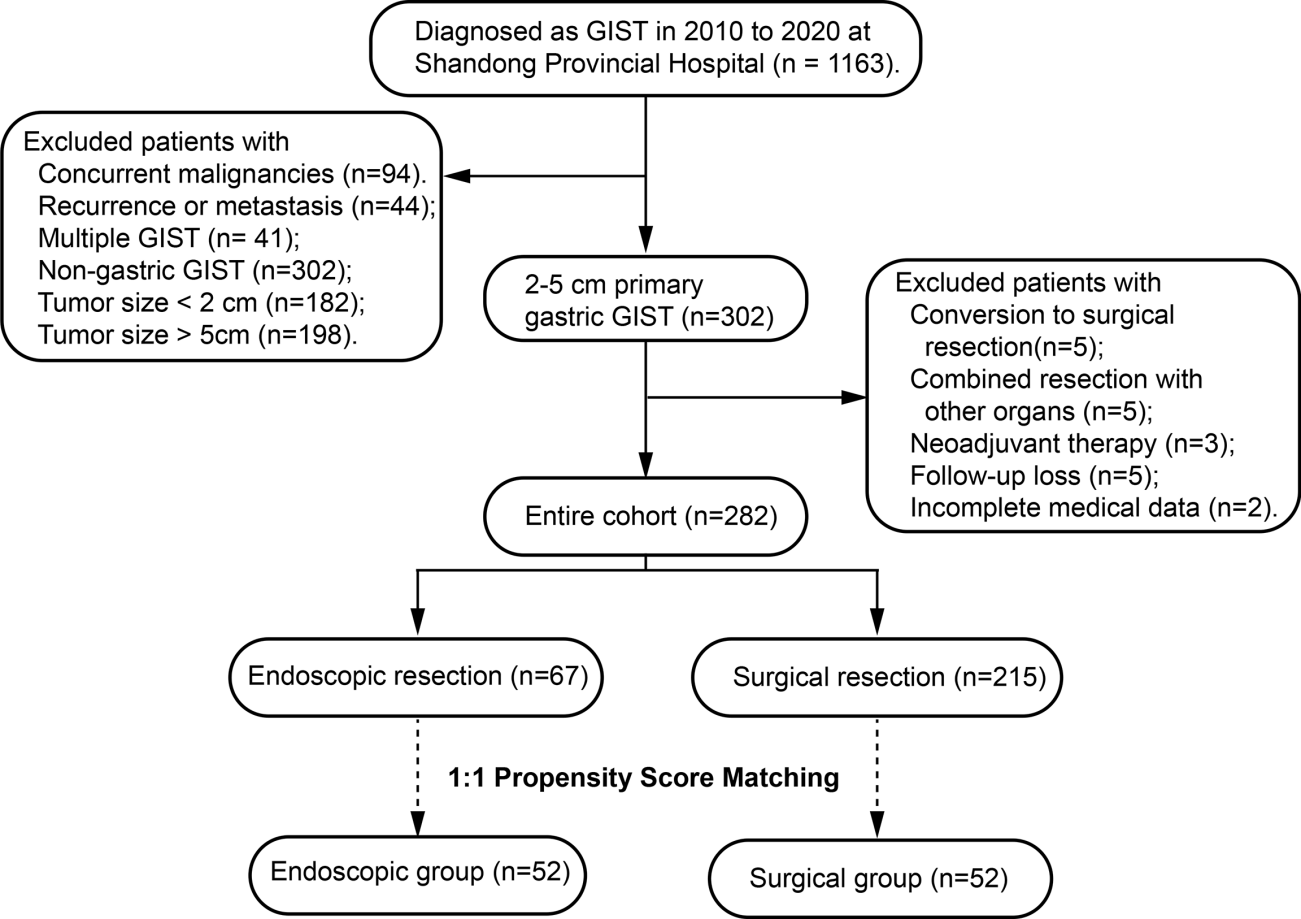
Flow chart of this study.

A total of 1163 consecutive patients diagnosed with GISTs and undergoing resection at Shandong Provincial Hospital between March 2010 and January 2020 were initially pooled. Among them, 302 were classified as having 2-5 cm primary GISTs in our institution **(** **Figure 1** **)**. All oncologic resections with curative intent were performed by senior experts to reach the rigorous standard at our institution.

Computed tomography (CT) examination was used to exclude adjacent organ infiltration and metastases before surgery. In addition, gastroscopy or ultrasound gastroscopy was performed to determine the location and size of the gastric GISTs. Cardiopulmonary examinations were routinely performed to assess tolerance to surgery.

Patients with the following criteria were included: (1) age over eighteen years; (2) pathological diagnosis of gastric GISTs; (3) tumour size 2–5 cm in diameter; (4) no evidence of recurrent GISTs or distant metastasis before treatment; and (5) American Society of Anesthesiology (ASA) score ≤ 3.

Patients having the following criteria were excluded: (1) any previous or concurrent malignancies; (2) underwent the first operation at another institution; (3) endoscopic resection conversion to surgical resection; (4) combined resection with other organs; (5) received neoadjuvant therapy; (6) severely missing or illegible medical data; and (7) missing follow-up data.

Finally, 282 patients with regular follow-up were included and analysed in the entire cohort. The follow-up was performed every 3 months for the first 3 years, then every 6 months up to 5 years, and then every year or until death. in the following years. The latest follow-up date was updated to December 2020.

### Data Collection

Following clinicopathological characteristics of patients were routinely collected from database of GISTs, including age, sex, body mass index (BMI), comorbidities, chief complaint, tumour location, tumour size, growth type, tumour shape, tumour origin, ulceration or high-risk imageology features, mitotic index (per 50 high power field), modified NIH (National Institutes of Health) risk category, surgical and endoscopic methods, estimated blood loss, operation time, resection margin, adverse events, time to liquid diet, hospitalization time, postoperative imatinib, immunohistochemistry (IHC) result, haematological indices and so on. The patients’ BMIs were classified into the following categories: < 18.5, 18.5–24.9 and ≥ 25 kg/m^2^, based on the World Health Organization (WHO) classification standards. The comorbidities analysed in this study comprised hypertension, diabetic mellitus, anaemia, pulmonary disease (asthma, pneumonia, chronic obstructive pulmonary disease, etc.), heart disease (arrhythmia, coronary atherosclerotic heart disease, etc.), liver disease (hepatitis, cirrhosis, etc.), renal disease (nephritis, chronic kidney disease, etc.) and central nervous system disease (cerebrovascular disease, neurodegenerative disease, etc.).

The primary outcome was progression-free survival (PFS), which was defined as the interval between the date of resection and confirmed disease progression or death. Patients were censored at the date of the last follow-up without the above event.

### Surgical and Endoscopic Resection

The surgical resection method is determined mainly based on the evaluation of tumour location and patient’s condition. In recent years, laparoscopic wedge resection has been the main surgical method. When the tumour was located adjacent to the pylorus or oesophagogastric junction, distal, proximal or gastrectomy was performed. Open surgery was usually performed when the adhesion was severe or the tumour location was inappropriate, and it was also performed in the early years.

For the endoscopic resection group, 5 types of endoscopic procedures were performed, including endoscopic submucosal dissection (ESD), endoscopic submucosal excavation (ESE), endoscopic full-thickness resection (EFR) and submucosal tunnelling endoscopic resection (STER). The method of endoscopic resection was also selected according to the characteristics of the tumour.

All resection procedures were performed by experts with similarly high levels of experience.

### Statistical Analysis

According to the expected values, categorical variables were analysed by Pearson’s chi-squared test or Fisher’s exact test. Continuous variables were compared by utilizing the Mann–Whitney *U*-test, which are presented as medians and interquartile ranges (IQRs) and as the mean ± standard deviation (SD). The Kaplan–Meier method and log-rank test were performed to conduct survival analyses and evaluate differences in survival time, respectively. A Cox proportional hazards model was used to perform univariate and multivariate analyses. Univariate analysis was primarily performed, and variables with *P* < 0.1 were subsequently computed into multivariate analysis to determine the independent prognostic factors. Hazard ratios (HRs) with their 95% confidence intervals (CIs) were also derived. Statistical significance was considered when *P* < 0.05.

R software version 3.5.3 (The R Foundation for Statistical Computing, Vienna, Austria) and SPSS 26.0 (IBM SPSS Statistics, Armonk, NY: IBM Corp) were used in this study.

### Propensity Score Matching

Propensity score matching was performed due to the inhomogeneous distribution of several baseline characteristics and uneven group sizes between SR and ER. First, the endoscopic resection group was regressed as a dependent variable on relevant baseline parameters, and a logistic regression model was used to calculate a propensity score. The propensity score matching ratio was set to a 1:1 ratio to minimize the differences due to age, sex, comorbidities, BMI, tumour location, tumour size, mitotic index and growth type with the nearest neighbour method. The assessment of propensity score matching is also shown (**Supplemental Figure 1**).

## Results

### Patient Characteristics

As shown in the flow chart (**Figure 1**), of the 282 consecutive patients who pooled into the entire cohort at our institution between March 2010 and January 2020, 67 patients in the ER group and 215 patients in the SR group were included.

The clinical characteristics are described in detail (**Table 1**). Before matching, the baseline characteristics of the patients, such as age, sex, BMI and comorbidities, were similar between the two groups. Regarding tumour characteristics, there were statistically significant differences in tumour size (*P*<0.001), tumour location (*P*=0.024), mitotic index (*P*=0.048), modified NIH risk category (*P*<0.001) and growth type (*P*=0.038). Regarding the clinicopathological characteristics of the tumour (**Supplemental Table 1**), significant differences were observed in ulceration (*P*<0.001) and high-risk imaging features (*P*<0.001), but there was no significant difference in the immunohistochemical results. Among the blood indicators, some were also found to be significantly different (**Supplemental Table 2**).

**Table 1:** Baseline clinicopathologic characteristics of SR and ER group in the entire cohort and after propensity score matching.

Parameters	Entire cohort (before matching)		*P* value	Propensity score matched cohort		*P* value
	SR, n (%)	ER, n (%)		SR, n (%)	ER, n (%)	
All cases	215	67		52	52	
Age (years)			0.341			0.433
≤ 60	98	35		24	28	
> 60	117	32		28	24	
Gender			0.369			0.694
Male	102	36		23	25	
Female	113	31		29	27	
BMI (kg/m^2^)			0.111			0.540
BMI < 18.5	7	5		1	5	
18.5 ≤ BMI < 25	95	35		24	27	
BMI ≥ 25	113	27		27	20	
Comorbidities*			0.591			0.842
Present	99	27		22	21	
Absent	126	40		30	31	
Location			**0.024**			0.247
Fundus	6	2		2	0	
Cardia	104	44		32	33	
Body	77	19		13	19	
Antrum	28	2		5	2	
Tumor size			**<0.001**			0.343
Mean ± SD	3.87 ± 0.91	2.58 ± 0.60		2.84 ± 0.70	2.72 ± 0.61	
Median (IQR)	4.0 (3.0-4.6)	2.5 (2.0-3.0)		2.6 (2.3-3.0)	2.5 (2.35-3.0)	
Mitotic index (per 50 HPF)			**0.048**			0.112
0-5	171	61		50	46	
6-10	33	6		1	6	
>10	11	0		1	0	
Modified NIH risk			**<0.001**			0.112
Very low	9	12		5	7	
Low	155	49		45	39	
Intermediate	41	6		1	6	
High	10	0		1	0	
Growth type			**0.038**			0.791
Intraluminal	160	58		44	43	
Extraluminal	55	9		8	9	

Bold values indicate P<0.05. BMI, Body Mass Index; SR, Surgical resection; ER, Endoscopic resection.

*Comorbidities: comprised of hypertension, diabetic mellitus, anaemia, pulmonary disease (asthma, pneumonia, chronic obstructive pulmonary disease, etc.), heart disease (arrhythmia, coronary atherosclerotic heart disease, etc.), liver disease (hepatitis, cirrhosis, etc.), renal disease (nephritis, chronic kidney disease, etc.) and central nervous system disease (cerebrovascular disease, neurodegenerative disease, etc.).

After propensity score matching, there were no significant differences among all variables, and all baseline and tumour characteristics of 52 patients in the ER group were compared with 52 patients in the SR group. Supplemental clinicopathological characteristics and blood indicators were also compared, and there was a difference only in the high-risk imaging features (*P*=0.016).

The resection methods of the SR and ER groups in the entire cohort and after propensity score matching are also summarized (**Supplemental Table 3**). The GISTs of 5 patients were unsuccessfully resected in the first attempt at endoscopic resection and switched to surgical resection. We did not include these patients in entire cohort, but they are very important for evaluating resection methods, which we also summarized (**Table 2**). In addition, tumours with transverse diameters >3.5 cm were very difficult to remove completely through the cardia. Patients with tumours larger than 3.5 cm were also included in the analysis presented in **Table 3**.

**Table 2:** Clinical characteristics of patients who were converted to surgical resection.

Case no.	Age	Gender	Location	Size (cm)	Method	Procedure time (min)	Origin	Reason	Hospital stay (day)
1	39	Female	Fundus	3	ESD	170	Muscularis propria	Bleeding	7
2	73	Male	Fundus	3.5	ESD	180	Muscularis propria	Bleeding	7
3	49	Female	Body	3.5	ESE	120	Muscularis propria	Bleeding	8
4	61	Female	Antrum	3	ESD	100	Muscularis propria	Resection difficulty	13
5	54	Male	Fundus	2.5	ESD	100	Muscularis propria	Bleeding	7

**Table 3:** Clinical characteristics of patients with tumours larger than 3.5 cm and undergoing endoscopic resection.

Case no.	Age	Gender	Location	Size (cm)	Mitoses per 50 HPFs	High-risk feature	Method	En bloc resection	Resection margin	Procedure time (min)	Hospital stay (day)	Adverse events	Follow-up (month)	Survival
1	70	Female	Fundus	4	3	/	ESD	No	R0	75	6	Perforation	104.8	Alive
2	73	Female	Fundus	3.5	<5	Ulceration	ESE	Yes	R0	55	5	/	71.1	Alive
3	67	Female	Fundus	4.5	<5	/	ESD	No	R0	55	4	/	33.3	Alive
4	70	Female	Body	4	3	Irregular shape	EFR	Yes	R0	90	5	/	32.6	Alive
5	61	Male	Fundus	4	3	Heterogeneity	EFR	No	R0	120	6	/	27.5	Alive
6	76	Male	Fundus	3.5	1~2	/	ESE	No	R0	45	5	/	66.4	Alive

### Comparison of Short−Term Outcomes

The comparison of short-term outcomes between the ER and SR groups is summarized in **Table 4**. Before PSM, the operation time (*P*=0.001), time to liquid diet (*P*=0.001) and postoperative hospital stays (*P*=0.001) were significantly shorter in the ER group than in the SR group. No significant differences were observed in the achievement of a negative resection margin. Furthermore, the ER group also showed significantly less bleeding (*P*=0.037), although there were deficiencies in en bloc resection (*P*<0.001) and an increased number of adverse events (*P*=0.001) compared with the SR group. The clinical characteristics and treatments of patients with adverse events are also summarized (**Table 5**).

**Table 4:** Perioperative characteristics and long-term outcomes of SR and ER group in the entire cohort and after propensity score matching.

Parameters	Entire cohort (before matching)		*P* value	Propensity score matched cohort		*P* value
	SR, n (%)	ER, n (%)		SR, n (%)	ER, n (%)	
All cases	215	67		52	52	
Operate time (min)			**0.001**			**0.005**
Mean ± SD	104.5 ± 41.5	85.4 ± 41.4		110.0 ± 41.1	86.5 ± 42.7	
Median (IQR)	95 (75-130)	75 (50-120)		100 (16-130)	70 (50-142)	
En bloc resection			**<0.001**			**<0.001**
Yes	215	55		52	41	
No	0	12		0	11	
Estimated blood loss (ml)			**0.037**			0.741
≤50	172	61		46	48	
>50	43	6		6	4	
Resection margin			1.000			1.000
R0	215	66		52	51	
R1/R2	0	1		0	1	
Time to liquid diet (days)			**0.001**			0.055
Mean ± SD	3.41 ± 1.23	2.85 ± 1.20		3.35 ± 0.97	2.92 ± 1.23	
Median (IQR)	3 (2-4)	3 (2-4)		3 (3-4)	3 (2-4)	
Postoperative hospital stays (days)			**<0.001**			**0.004**
Mean ± SD	6.85 ± 2.52	5.46 ± 1.79		6.44 ± 1.89	5.38 ± 1.76	
Median (IQR)	6 (5-8)	5 (4-6)		6 (5-7)	5 (4-6)	
Adverse events			**0.001**			**0.027**
Present	6	9		0	6	
Absent	209	62		52	46	
Imatinib treatment			0.088			1.000
Yes	30	4		4	3	
No	185	63		48	49	
Recurrence	12	5		0	3	

Bold values indicate P<0.05.

HPF, High Power Field; SD, Standard Deviation; IQR, Interquartile Range; NIH, National Institutes of Health; SR, Surgical resection; ER, Endoscopic resection.

**Table 5:** Clinical characteristics and treatments of patients with adverse events.

Case no.	Age	Gender	Location	Size (cm)	Method	Adverse events	Procedure time (min)	Hospital stay (days)	Treatment
1	70	Female	Fundus	4	ESD	Perforation	75	6	Laparoscopic repair
2	60	Male	Fundus	2	ESD	Perforation	150	9	Laparoscopic repair
3	44	Male	Fundus	2	ESE	Perforation	150	10	Laparoscopic repair
4	60	Female	Fundus	5	Lap wedge	Bleeding	70	14	Blood transfusion
5	58	Female	Body	2.5	ESE	Perforation	150	6	Laparoscopic repair
6	64	Female	Fundus	2.4	EFR	Peritonitis	50	8	Antibiotic treatment
7	52	Female	Fundus	2	EFR	Peritonitis	30	5	Antibiotic treatment
8	41	Male	Cardia	2	STER	Subcutaneous emphysema	75	5	Closed drainage
9	61	Female	Body	2.3	EFR	Bleeding	45	5	Endoscopic haemostasis
10	69	Male	Body	2.7	EFR	Perforation	150	9	Laparoscopic repair
11	55	Female	Fundus	5	Open gastrectomy	Pleural effusion	100	9	Thoracentesis and drainage
12	60	Male	Fundus	3.5	Open gastrectomy	Remnant Gastritis	155	10	Conservative
13	51	Male	Antrum	4.5	Open gastrectomy	Jaundice	80	14	Conservative
14	61	Female	Body	3.5	Lap wedge	Stricture	85	6	Conservative
15	53	Male	Body	4	Lap gastrectomy	Bleeding	90	7	Exploratory laparotomy

ESD, Endoscopic Submucosal Dissection; ESE, Endoscopic Submucosal Excavation; EFR, Endoscopic Full-Thickness Resection; STER, Submucosal Tunneling Endoscopic Resection; Lap, laparoscopic.

After matching, the operation time (*P*=0.005) and postoperative hospital stay (*P*=0.005) were also significantly longer in the SR group than in the ER group. However, there were no significant differences in blood loss (*P*=0.741), resection margin (*P*=1.000) or time to liquid diet (*P*=0.055). Statistical differences were also seen in en bloc resection (P<0.001) and adverse events (*P*=0.027).

### Comparison of Long−Term Outcomes

**Figure 2:**
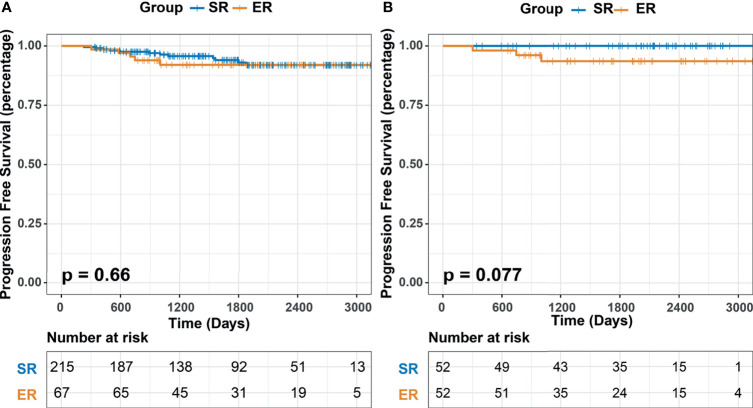
Kaplan–Meier survival analysis of progression-free survival.

The median follow-up time of the entire matched cohort was 1660 days (IQR, 950-2399), and the 1-, 3- and 5-year PFS rates were 98.94%, 95.39% and 94.33%, respectively. Prior to the last follow-up, a recurrence of GISTs occurred in 12 patients in the SR group (5.58%, 12/215) and 5 patients in the ER group (7.46%, 5/67), but the difference was not significant (*P*=0.660) (**Figure 2**). For the propensity score matched cohort, significant differences could also not be observed (*P*=0.077).

We continued to utilize univariate analysis to identify the mitotic index (*P*=0.004) and ulceration (*P*=0.001) as prognostic factors for PFS (**Table 6**). On multivariate analysis, mitotic index (HR=2.823, 95% CI=0.083-9.122, *P*=0.083; HR=6.385, 95% CI=1.733-23.523, *P*=0.005, respectively) and ulceration (HR=4.909, 95% CI=1.873-12.867, *P*=0.001) were eventually identified as independent prognostic factors.

**Table 6:** Univariate and multivariate of the clinicopathological factors for progression-free survival.

Characteristics	Univariate analysis		Multivariate analysis	
	HR (95% CI)	*P*-value	HR (95% CI)	*P*-value
Age (year)				
≤60	Reference			
>60	1.810 (0.669-4.896)	0.243		
Gender				
Female	Reference			
Male	0.745 (0.283-1.957)	0.550		
Comorbidities*				
Absent	Reference			
Present	0.915 (0.348-2.406)	0.858		
Tumor size (cm)	1.505 (0.924-2.452)	0.101		
Mitotic index (per 50 HPF)		**0.004**		**0.012**
0-5	Reference		Reference	
6-10	2.638 (0.824-8.448)	0.102	2.823 (0.874-9.122)	0.083
>10	8.265 (2.256-30.285)	**0.001**	6.385 (1.733-23.523)	**0.005**
En bloc resection				
Yes	Reference			
No	2.945 (0.674-12.909)	0.151		
Growth type				
Intraluminal	Reference			
Extraluminal	0.523 (0.120-2.291)	0.390		
Shape				
Regular	Reference			
Irregular	1.866 (0.427-8.166)	0.407		
Ulceration				
Present	Reference		Reference	
Absent	5.254 (2.027-13.620)	**0.001**	4.909 (1.873-12.867)	**0.001**
High-risk imageology features†				
Present	Reference			
Absent	1.597 (0.608-4.196)	0.342		
Group				
SR	Reference			
ER	1.265 (0.446-3.592)	0.659		

Bold values indicate P<0.05.

HPF, High Power Field; SR, Surgical resection; ER, Endoscopic resection.

*Comorbidities: comprised of hypertension, diabetic mellitus, anaemia, pulmonary disease (asthma, pneumonia, chronic obstructive pulmonary disease, etc.), heart disease (arrhythmia, coronary atherosclerotic heart disease, etc.), liver disease (hepatitis, cirrhosis, etc.), renal disease (nephritis, chronic kidney disease, etc.) and central nervous system disease (cerebrovascular disease, neurodegenerative disease, etc.).

†High-risk imageology features: heterogeneity, hyperechoic foci, or cystic spaces.

### Subgroup Analysis

We found that in the cohort after propensity score, most of the tumour diameters were between 2 and 3 cm. Then, we continued to compare surgical and endoscopic resection for patients with 2-3 cm gastric gastrointestinal stromal tumours (**Supplemental Table 4**).

After propensity score matching, there were no significant differences among all characteristic variables. For short−term outcomes (**Supplemental Table 5**), operation time (*P*=0.062), estimated blood loss (*P*=0.485) and adverse events (*P*=0.056) were comparable. Although the ER group still had significant advantages in time to liquid diet (*P*=0.043) and postoperative hospital stays (*P*=0.031), the achievement of en bloc resection (*P*=0.026) still needs to be improved.

In recent years, with the development of medical devices and the advancement of surgical techniques, laparoscopic resection and advanced endoscopic resection, including ESE, EFR and STER, have gradually become the primary choices for the treatment of muscularis propria tumours. Therefore, we compared laparoscopic and advanced endoscopic resection for gastrointestinal stromal tumours originating from the muscularis propria (**Supplemental Table 6**).

There were no significant differences among all characteristic variables after propensity score matching. The ER group still had a significantly shorter operation time (*P*=0.002) and postoperative hospital stay (*P*=0.009). The two groups were equal in blood loss (*P*=1.000), resection margin (*P*=1.000), time to liquid diet (*P*=0.072) and adverse events (*P*=0.237). However, in the ER group, there was a risk of difficult resection and recurrence (**Supplemental Table 7**).

For gastric GISTs larger than 3 cm, we also tried to make some reports descriptively (**Supplemental Table 8**). Although the patients in the SR group were older and had a higher rate of malignancy, surprisingly, there were no statistical differences in the operation time, blood loss, liquid diet time, and postoperative hospital stay. Of course, this has not yet taken into account that patients in the ER group have failed resections and converted to surgical resections (**Supplemental Table 9**). Although we also tried propensity score matching, there were too few patients in the ER group to obtain convincing results.

## Discussion

The management of small GISTs is still debated between surgeons and endoscopists (13, 14). In recent years, many studies have demonstrated that ER could be an effective, safe, and feasible therapeutic method for small gastric GISTs (15–17). Currently, there are also some studies aimed at comparing the clinical outcomes of ER and SR (18, 19). However, we found that the mean size of tumours was far below 5 cm, and the majority of them were even below 2 cm, which may have biased the results in favour of ER. At the same time, there are few descriptions of tumour origin and high-risk characteristics in existing studies. Therefore, the comparison of outcomes is often affected by clinical judgement; for example, patients with extraluminal tumours or imaging findings indicating highly malignant potential often undergo surgical resection.

In the present study, we performed propensity score matching using seven covariates, including age, sex, comorbidities, BMI, tumour location, tumour size, mitotic index and growth type, to compare the safety and efficacy of ER and SR for 2-5 cm gastric GISTs. Individual patient information that requires special instructions is also summarized.

Regarding short-term outcomes, ER had certain advantages over SR, such as shorter operation time (86.5 ± 42.7 *vs* 110.0 ± 41.1 minutes, *P*=0.005) and shorter postoperative hospital stays (5.38 ± 1.76 *vs* 6.44 ± 1.89 days, *P*=0.027). In our hospital, patients were routinely kept several days to ensure the recovery of gastrointestinal function and no postoperative complications. However, in recent years, laparoscopic resection has gradually become the recommended surgical method for GISTs in favourable anatomic locations, which is associated with shorter operation times and hospital stays. In addition, with the development and promotion of enhanced recovery after surgery (ERAS), the postoperative hospital stays after surgeries have been greatly reduced (20). In the subgroup analysis of LR and ER, we found that for 2-3 cm GISTs, the difference in operation time between the two groups was not statistically significant. For similar reasons, we found that the estimated blood loss in the ER group and the SR group were similar. In terms of time to liquid diet, there was a significant difference only in 2-3 cm GISTs.

Moreover, our data show that the incidence rate of adverse events in the ER group was relatively higher than that in the SR group. For the ER group, perforation was common and increasingly considered a minor adverse event due to endoscopic clipping and suturing techniques. In this study, most endoscopic perforation patients were successfully repaired endoscopically without conversions to emergency surgical operation, and we recorded only perforations that required surgical intervention. A total of four patients had macroperforation during endoscopic resection and underwent emergency surgery immediately.

Bleeding is also a common adverse event. One patient had bleeding after endoscopy resection and underwent a second endoscopic haemostasis procedure. In addition, there were 2 cases of delayed bleeding after surgery: one patient underwent exploratory surgery, and the other was treated conservatively after blood transfusion. All patients were fully discharged from the hospital. In our study, anastomotic stricture occurred in a patient one month after laparoscopic resection, and conservative treatment was performed.

Moreover, two patients suffered from peritonitis and recovered after fasting, nutritional support, antibiotic treatment and so on. In addition, some uncommon adverse events were recorded, such as jaundice and subcutaneous emphysema. Ultimately, all patients recovered and were discharged from the hospital after proper treatment.

In addition, due to massive intraoperative bleeding in 4 patients and resection difficulty in 1 patient, five GISTs were unsuccessfully resected in the first attempt at endoscopic resection. Therefore, surgeons had to perform surgical operations immediately to remove the tumour completely to achieve R0 resection.

The risk of tumour rupture and remnants is a major concern for ER. The R0 resection rates of ER are slightly lower than those of SR. However, a meta-analysis performed by our team showed that R1 resection did not influence the recurrence of GISTs.Tumour rupture is significantly associated with the occurrence of R1 resection and may be a confounder of R1 resection in GISTs (21). Tumour rupture could be caused by the removal of GISTs larger than 3 cm under peroral endoscopy. Due to the limited operating space and visual field, it might be difficult for endoscopists to maintain the integrity of a 2-5 cm gastric GIST. Although some large GISTs were resected in one piece (en bloc resection) successfully, they had to be divided into small pieces for removal. The en bloc resection rate was 82.09% (55/67) in our study. Considering that the tumours we included were all larger than 2 cm, this rate is acceptable. Of the 12 patients, two recurrences were noted during follow-up, and both were diagnosed with moderate-risk GISTs. Du et al. evaluated risk factors associated with piecemeal resection and demonstrated that large size and irregular shape may play a role (22).

Considering the risk of tumour rupture, tumour remnants, perforation, and bleeding, some experts consider the indications for endoscopic treatment for GISTs to be as follows: tumour size less than 3 cm, mainly an intraluminal growth pattern, a clear tumour boundary and uniform texture, and no symptoms of invasion and metastasis to other locations (23). Due to the above restrictions, ER is not currently recommended as a routine treatment for GISTs, and SR is still considered the standard treatment for 2-5 cm GISTs by most guidelines.

Regarding long-term outcomes, there are currently still insufficient studies. After following up with the patients in our hospital for the last decade, we found that the recurrence rate did not differ significantly between the those undergoing the two techniques. Our findings are consistent with the published articles thus far (23, 24).

We further performed univariate and multivariate analyses, and mitotic index (HR=2.823, 95% CI=0.083-9.122, P=0.083; HR=6.385, 95% CI=1.733-23.523, P=0.005, respectively) and ulceration (HR=4.909, 95% CI=1.873-12.867, P=0.001) were eventually identified as independent prognostic factors of PFS, which was partly similar to the prediction model for high malignancy potential of Yang et al (25 **).**

In the subgroup analysis, we also obtained some inspiring findings. For 2-3 cm gastric GISTs, although the advantage of the ER in operation time is no longer significant, the number of adverse events do not seem to be significantly different. ER might be an effective strategy for improving postoperative recovery without increasing the risk of surgery and recurrence for these selected GISTs.

SR and ER comprise two alternative options for the treatment of GISTs originating from the muscularis propria (MP) layer, and there is currently no study comparing the two methods. Currently, ESE, EFR and STER are the three main endoscopic methods to resect these GISTs (26). Based on the limited information available, we found that ER still has advantages in terms of operating time and hospital stay, but there is no significant advantage in liquid diet. Of course, the most surprising thing is that these advanced endoscopic techniques are excellent in reducing the occurrence of adverse events.

Recently, the nonexposure simple suturing endoscopic full-thickness resection (NESS-EFTR) method was developed to avoid tumour exposure to the peritoneal cavity (27). In the future, it will be more exciting to look forward to the emergence of technologies that can avoid tumour exposure under complete endoscopic full-thickness resection. There are an increasing number of options available for doctors to choose, but these multiple options also bring difficulties to the evaluation of the most suitable method (28–30).

Our results indicate that SR is safer and results in fewer intraoperative adverse events than ER. It might be appropriate to determine the method of resection according to tumour location and mucosal ulceration. Regardless of the technique chosen, detailed routine physical examination should be carried out, such as endoscopic ultrasonography (EUS) and abdominal CT, to assess the location, border, size, origin and even blood supply of the lesions (6).

There are many highlights of our research. The general distribution of tumour size was usually not clear and precise, as stated in previous studies. In addition, previous studies did not pay attention to the growth type of tumours, and the malignant potential was not well stated, which may lead to some biases. We provided a detailed and comprehensive description of the tumours included in the study and applied PSM to ensure suitable randomization in the evaluation of short- and long-term outcomes.

Our study also has several limitations. First, it is difficult for a single centre to enrol a very large cohort for GISTs, a relatively rare neoplasm. To the best of our knowledge, our cohort is one of the largest samples in the relevant field. Second, we excluded five patients who had additional surgery because of incomplete resection of the GISTs after ER, which may have biased the results in favour of ER. Third, with a small number of events, it was difficult to have good reliability and validity. Propensity match score could also not eliminate all biases of the study. Last, as a retrospective study, some intraoperative and postoperative information may not be assiduously observed and recorded in detail. We will carry out multi-centre trials for comparison in the future.

## Conclusions

In conclusion, ER might be a safe and feasible method that is comparable to SR for the treatment of 2-3 cm gastric GISTs. SR is still considered the standard treatment for 3-5 cm gastric GISTs, while the intraoperative and postoperative information of ER should be recorded in detail and closely evaluated. Surgical resection is recommended if the tumour has a high mitotic index or mucosal ulceration. All patients should be carefully evaluated preoperatively to select the most appropriate resection procedure, and more prospective multi-centre random control trials with long-term follow-up are warranted to determine the best management of GISTs in the future.

## Data Availability Statement

The raw data supporting the conclusions of this article will be made available by the authors, without undue reservation.

## Ethics Statement

This study was approved by the Ethics Committee of Shandong Provincial Hospital (SWYX: No. 2021-035). Written informed consent for participation was not required for this study in accordance with the national legislation and the institutional requirements.

## Author Contributions

All authors helped to perform the research; HW and HL were involved in the conception and design; MF and WX were involved in the follow-up of patients; QX and RZ were involved in preliminary medical record screening and entry; LS was involved in the verification of the included data; CL and JC were involved in the drafting of the paper or revising it critically for intellectual content; JL and LL were involved in the final approval of the version to be published. All authors listed have made a substantial, direct, and intellectual contribution to the work, and approved it for publication.

## Funding

Supported by Key Research and Development Program of Shandong Province (No.2019JZZY010104; No.2019GSF108146); Special Foundation for Taishan Scholars Program of Shandong Province (No.ts20190978); Academic promotion programme of Shandong First Medical University (No. 2019QL021): Science and Technology Development Plan of Jinan 202019082.

## Conflict of Interest

The authors declare that the research was conducted in the absence of any commercial or financial relationships that could be construed as a potential conflict of interest.

## Publisher’s Note

All claims expressed in this article are solely those of the authors and do not necessarily represent those of their affiliated organizations, or those of the publisher, the editors and the reviewers. Any product that may be evaluated in this article, or claim that may be made by its manufacturer, is not guaranteed or endorsed by the publisher.

## Supplementary Material

Supplementary Figure 1 Evaluation of propensity score matching. **(A)** Jitter plot of individual cases; **(B)** Line plot of individual differences; **(C)** Histogram of propensity score; **(D)** Dot plot of standardized mean differences.

## Abbreviations

GISTs, Gastrointestinal Stromal Tumors; GI, gastrointestinal; NCCN, National Comprehensive Cancer Network; ESMO, European Society for Medical Oncology; SR, Surgical Resection; ER, Endoscopic Resection; LR, Laparoscopic Resection; CT, Computed Tomography; BMI, Body Mass Index; ASA, American Society of Anesthesiology; ESD, Endoscopic Submucosal Dissection; ESE, Endoscopic Submucosal Excavation; EFR, Endoscopic Full-Thickness Resection; STER, Submucosal Tunneling Endoscopic Resection; HPF, High Power Field; SD, Standard Deviation; IQR, Interquartile Range; CI, Confidence Interval; HR, Hazard ratios; NIH, National Institutes of Health; PFS, Progression-free Survival; IHC, Immunohistochemistry; ERAS, Enhanced Recovery After Surgery; MP, muscularis propria; NESS-EFTR, Non-Exposure Simple Suturing Endoscopic Full-Thickness Resection; EUS, Endoscopic Ultrasonography; NA, Not Available.
