# Supplementary figures and images for: Surgical Resection Is Still Better Than Endoscopic Resection for Patients With 2-5 cm Gastric Gastrointestinal Stromal Tumours: A Propensity Score Matching Analysis
Source: Front Oncol. 2021 Sep 15;11:737885. doi: 10.3389/fonc.2021.737885 (PMC8479163; doi:10.3389/fonc.2021.737885)

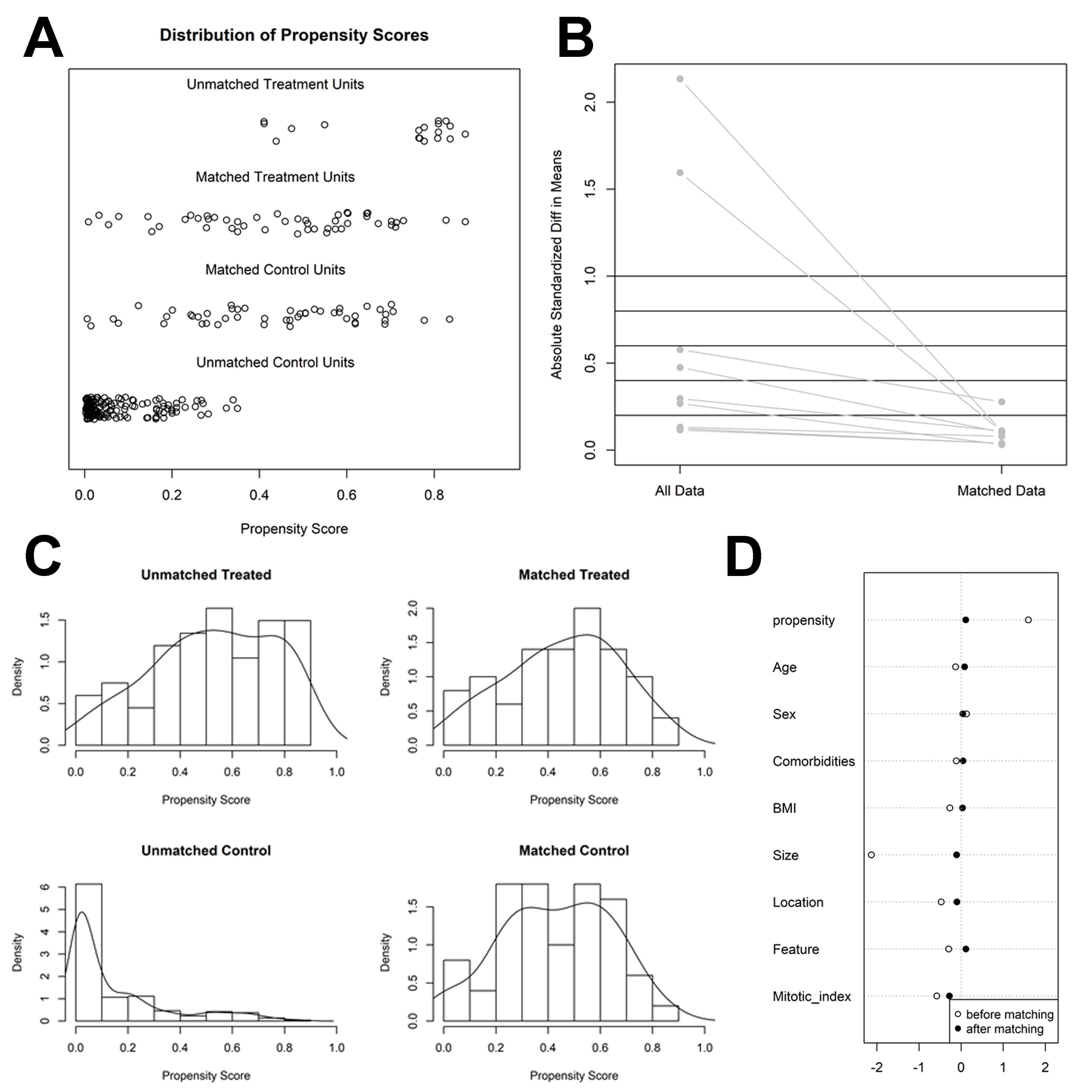

Supplement: Supplementary Figure 1 — Evaluation of propensity score matching. (A) Jitter plot of individual cases; (B) Line plot of individual differences; (C) Histogram of propensity score; (D) Dot plot of standardized mean differences. [file Image_1.tif]
