# Supplementary material for: Surgical Resection Is Still Better Than Endoscopic Resection for Patients With 2-5 cm Gastric Gastrointestinal Stromal Tumours: A Propensity Score Matching Analysis
Source: Front Oncol. 2021 Sep 15;11:737885. doi: 10.3389/fonc.2021.737885 (PMC8479163; doi:10.3389/fonc.2021.737885)
Supplement: Supplementary file 2 [file DataSheet_1.zip › Table_1.docx]

| Parameters | Entire cohort (before matching) | | *P*  value | Propensity score matched cohort | | *P*  value |
| --- | --- | --- | --- | --- | --- | --- |
|  | SR, n (%) | ER, n (%) |  | SR, n (%) | ER, n (%) |  |
| All cases | 215 | 67 |  | 52 | 52 |  |
| IHC |  |  |  |  |  |  |
| CD117 (+) | 212/215 | 67/67 | 1.000 | 50/52 | 52/52 | 0.495 |
| CD34 (+) | 212/215 | 67/67 | 1.000 | 51/52 | 52/52 | 1.000 |
| DOG-1 (+) | 209/215 | 67/67 | 0.341 | 51/52 | 52/52 | 1.000 |
| SMA (+) | 38/202 | 17/65 | 0.219 | 12/50 | 13/50 | 0.817 |
| S-100 (+) | 9/195 | 1/60 | 0.460 | 1/49 | 0/46 | 1.000 |
| Desmin (+) | 2/67 | 3/24 | 0.112 | 1/19 | 2/18 | 1.000 |
| Ki-67* | 3 (2-5) | 3 (2-5) | 0.327 | 3 (2-5) | 3 (2-5) | 0.120 |
| Shape |  |  | 0.971 |  |  | 0.437 |
| Regular | 199 | 61 |  | 50 | 47 |  |
| Irregular | 16 | 5 |  | 2 | 5 |  |
| Origin |  |  | 0.324 |  |  | 0.117 |
| Muscularis mucosal | 8 | 2 |  | 5 | 2 |  |
| Muscularis propria | 103 | 65 |  | 31 | 50 |  |
| NA | 104 | 0 |  | 16 | 0 |  |
| Ulceration |  |  | **<0.001** |  |  | 0.092 |
| Present | 50 | 2 |  | 8 | 2 |  |
| Absent | 165 | 65 |  | 44 | 50 |  |
| High-risk imageology features† |  |  | **<0.001** |  |  | **0.016** |
| Present | 121 | 17 |  | 27 | 15 |  |
| Absent | 94 | 50 |  | 25 | 37 |  |

**Supplemental Table 1**

**Supplemental clinicopathological characteristics of SR and ER group in the entire cohort and after propensity score matching.**

Bold values indicate P<0.05; *median (IQR); †High-risk imageology features: heterogeneity, hyperechoic foci, or cystic spaces.

SR: Surgical resection; ER: Endoscopic resection;
