# Supplementary material for: Surgical Resection Is Still Better Than Endoscopic Resection for Patients With 2-5 cm Gastric Gastrointestinal Stromal Tumours: A Propensity Score Matching Analysis
Source: Front Oncol. 2021 Sep 15;11:737885. doi: 10.3389/fonc.2021.737885 (PMC8479163; doi:10.3389/fonc.2021.737885)
Supplement: Supplementary file 2 [file DataSheet_1.zip › Table_2.docx]

| Parameters | Entire cohort (before matching) | | *P*  value | Propensity score matched cohort | | *P*  value |
| --- | --- | --- | --- | --- | --- | --- |
|  | SR, n (%) | ER, n (%) |  | SR, n (%) | ER, n (%) |  |
| All cases | 215 | 67 |  | 52 | 52 |  |
| Blood type |  |  | / |  |  | / |
| A | 66 | 10 |  | 17 | 9 |  |
| B | 56 | 26 |  | 15 | 22 |  |
| O | 61 | 12 |  | 12 | 7 |  |
| AB | 25 | 8 |  | 8 | 4 |  |
| NA | 7 | 11 |  | 0 | 10 |  |
| Blood indicators |  |  |  |  |  |  |
| WBC,10^9^/L * | 5.47 (4.57-6.45) | 5.13 (4.48-6.20) | 0.870 | 5.26 (4.34-6.24) | 5.12 (4.50-5.97) | 0.429 |
| RBC, 10^12^/L * | 4.37 (3.67-6.45) | 4.57 (4.28-4.90) | **<0.001** | 4.48 (4.25-4.73) | 4.54 (4.26-4.87) | 0.782 |
| HGB, g/L* | 130 (104.5-143) | 138 (131-152) | **<0.001** | 134 (120-146) | 136 (130-149) | 0.301 |
| PLT, 10^9^/L * | 235 (188.5-284) | 214 (175-243) | 0.075 | 231 (173-277) | 215 (175-243) | 0.141 |
| LYM, 10^9^/L * | 1.69 (1.34-2.07) | 1.65 (1.39-2.00) | 0.920 | 1.81 (1.46-2.07) | 1.64 (1.40-1.94) | 0.489 |
| MON, 10^9^/L * | 0.39 (0.30-0.48) | 0.37 (0.30-0.47) | 0.247 | 0.39 (0.33-0.43) | 0.37 (0.32-0.45) | 0.371 |
| NEU, 10^9^/L * | 3.14 (2.53-4.00) | 3.05 (2.35-3.97) | 0.869 | 3.00 (2.37-3.68) | 3.03 (2.37-3.94) | 0.399 |
| EO, 10^9^/L * | 0.08 (0.05-0.14) | 0.10 (0.05-0.16) | 0.695 | 0.10 (0.05-0.15) | 0.11 (0.06-0.16) | 0.348 |
| BASO, 10^9^/L * | 0.02 (0.01-0.03) | 0.02 (0.01-0.03) | **0.012** | 0.02 (0.01-0.03) | 0.02 (0.01-0.03) | 0.745 |
| AST, U/L* | 19 (17-23) | 19 (16-23) | 0.960 | 20 (17-26) | 19 (16-24) | 0.889 |
| ALT, U/L * | 15 (12-20) | 15 (13-19) | 0.423 | 17 (12-23) | 16 (12-20) | 0.181 |
| ALP, U/L * | 80 (66-95) | 78 (66-95) | 0.252 | 82 (64-95) | 80 (65-96) | 0.543 |
| PA, g/L* | 241 (194-280) | 243 (210-284) | 0.274 | 254 (207-301) | 240 (200-278) | 0.591 |
| ALB, g/L* | 40.6 (37.3-43.1) | 41.3 (39.4-45.7) | **0.008** | 40.9 (38.9-43.1) | 41.3 (39.2-45.8) | 0.632 |
| GLO, g/L * | 26.9 (23.8-29.4) | 26.7 (24.1-29.7) | 0.966 | 27.8 (24.5-30.6) | 26.7 (24.1-29.9) | 0.770 |
| TC, mmol/L* | 5.06 (4.26-5.76) | 5.21 (4.34-6.04) | 0.643 | 5.06 (3.97-6.00) | 5.26 (4.39-6.14) | 0.448 |

**Supplemental Table 2**

**Supplemental blood indicators of SR and ER group in the entire cohort and after propensity score matching.**

Bold values indicate P<0.05; *median (IQR).

SR: Surgical resection; ER: Endoscopic resection; IHC: Immunohistochemistry; WBC: White Blood Cell; RBC: Red Blood Cell; HGB: Hemoglobin; PLT: Platelet; LYM: Lymphocyte; MON: Monocyte; NEU: Neutrophil; EO: Eosinophil; BASO: Basophil; AST: Aspartate Aminotransferase; ALT: Alanine Aminotransferase; PA: Prealbumin; ALB: Albumin; GLO: Globulin; TC: Total Cholesterol; NA: Not Available.
