# Supplementary material for: Surgical Resection Is Still Better Than Endoscopic Resection for Patients With 2-5 cm Gastric Gastrointestinal Stromal Tumours: A Propensity Score Matching Analysis
Source: Front Oncol. 2021 Sep 15;11:737885. doi: 10.3389/fonc.2021.737885 (PMC8479163; doi:10.3389/fonc.2021.737885)
Supplement: Supplementary file 2 [file DataSheet_1.zip › Table_3.docx]

| Parameters | Entire cohort (before matching) | |  | Propensity score matched cohort | |
| --- | --- | --- | --- | --- | --- |
|  | SR, n (%) | ER, n (%) |  | SR, n (%) | ER, n (%) |
| All cases | 215 | 67 |  | 52 | 52 |
| Surgical methods |  |  |  |  |  |
| Open | 80 |  |  | 8 |  |
| Laparoscopic | 122 |  |  | 35 |  |
| LECS | 13 |  |  | 10 |  |
| Endoscopic methods |  |  |  |  |  |
| ESD |  | 16 |  |  | 14 |
| ESE |  | 28 |  |  | 20 |
| EFR |  | 20 |  |  | 17 |
| STER |  | 3 |  |  | 1 |

**Supplemental Table 3**

**Supplemental resection methods of SR and ER group in the entire cohort and after propensity score matching.**

SR: Surgical Resection; ER: Endoscopic Resection; LECS: Laparoscopic and Endoscopic Cooperative Surgery; ESD: Endoscopic Submucosal Dissection; ESE: Endoscopic Submucosal Excavation; EFR: Endoscopic Full-Thickness Resection; STER: Submucosal Tunneling Endoscopic Resection;
