# Supplementary material for: Surgical Resection Is Still Better Than Endoscopic Resection for Patients With 2-5 cm Gastric Gastrointestinal Stromal Tumours: A Propensity Score Matching Analysis
Source: Front Oncol. 2021 Sep 15;11:737885. doi: 10.3389/fonc.2021.737885 (PMC8479163; doi:10.3389/fonc.2021.737885)
Supplement: Supplementary file 2 [file DataSheet_1.zip › Table_5.docx]

| Parameters | Entire cohort (before matching) | | *P*  value | Propensity score matched cohort | | *P*  value |
| --- | --- | --- | --- | --- | --- | --- |
|  | SR, n (%) | ER, n (%) |  | SR, n (%) | ER, n (%) |  |
| All cases | 56 | 61 |  | 46 | 46 |  |
| Operate time (min) |  |  | **0.034** |  |  | 0.062 |
| Mean ± SD | 103.0±39.7 | 86.6±42.5 |  | 104.6±38.9 | 88.5±42.6 |  |
| Median (IQR) | 95 (75-130) | 75 (50-130) |  | 98 (75-130) | 75 (50-150) |  |
| En bloc resection |  |  | **0.006** |  |  | **0.026** |
| Yes | 56 | 53 |  | 46 | 40 |  |
| No | 0 | 8 |  | 0 | 6 |  |
| Estimated blood loss (ml) |  |  | 0.494 |  |  | 0.485 |
| ≤ 50 | 53 | 55 |  | 43 | 40 |  |
| > 50 | 3 | 6 |  | 3 | 6 |  |
| Resection margin |  |  | 1.000 |  |  | 1.000 |
| R0 | 56 | 60 |  | 46 | 45 |  |
| R1/R2 | 0 | 1 |  | 0 | 1 |  |
| Time to liquid diet (days) |  |  | **0.049** |  |  | **0.043** |
| Mean ± SD | 3.25±1.00 | 2.84±1.23 |  | 3.28±1.03 | 2.80±1.20 |  |
| Median (IQR) | 3 (2-4) | 3 (2-3.5) |  | 3 (2-4) | 3 (2-3.5) |  |
| Postoperative hospital stays (days) |  |  | **0.018** |  |  | **0.031** |
| Mean ± SD | 6.32±1.89 | 5.49±1.86 |  | 6.46±1.97 | 5.59±1.82 |  |
| Median (IQR) | 6 (5-8) | 5 (4-6) |  | 6 (5-8) | 5.5 (4-6) |  |
| Adverse events |  |  | **0.006** |  |  | 0.056 |
| Present | 0 | 8 |  | 0 | 5 |  |
| Absent | 56 | 53 |  | 46 | 41 |  |
| Imatinib treatment |  |  | 0.226 |  |  | 0.739 |
| Yes | 8 | 4 |  | 6 | 4 |  |
| No | 48 | 57 |  | 40 | 42 |  |
| Recurrence | 1 | 4 |  | 1 | 3 |  |

**Supplemental Table 5**

**Perioperative characteristics and long-term outcomes of SR and ER group of 2-3 cm GISTs in the entire cohort and after propensity score matching.**

Bold values indicate P<0.05.

HPF: High Power Field; SD: Standard Deviation; IQR: Interquartile Range; NIH: National Institutes of Health; SR: Surgical resection; ER: Endoscopic resection.
