# Supplementary material for: Surgical Resection Is Still Better Than Endoscopic Resection for Patients With 2-5 cm Gastric Gastrointestinal Stromal Tumours: A Propensity Score Matching Analysis
Source: Front Oncol. 2021 Sep 15;11:737885. doi: 10.3389/fonc.2021.737885 (PMC8479163; doi:10.3389/fonc.2021.737885)
Supplement: Supplementary file 2 [file DataSheet_1.zip › Table_7.docx]

| Parameters | Entire cohort (before matching) | | *P*  value | Propensity score matched cohort | | *P*  value |
| --- | --- | --- | --- | --- | --- | --- |
|  | LR, n (%) | ER, n (%) |  | LR, n (%) | ER, n (%) |  |
| All cases | 70 | 51 |  | 30 | 30 |  |
| Operate time (min) |  |  | **0.001** |  |  | **0.002** |
| Mean ± SD | 114.9±43.7 | 88.5±41.1 |  | 123.5±41.6 | 89.2±38.2 |  |
| Median (IQR) | 110 (79-150) | 75 (50-130) |  | 128 (80-151) | 88 (54-120) |  |
| En bloc resection |  |  | **0.002** |  |  | **0.024** |
| Yes | 70 | 44 |  | 30 | 24 |  |
| No | 0 | 7 |  | 0 | 6 |  |
| Estimated blood loss (ml) |  |  | 0.537 |  |  | 1.000 |
| ≤ 50 | 59 | 45 |  | 26 | 26 |  |
| > 50 | 11 | 6 |  | 4 | 4 |  |
| Resection margin |  |  | 0.421 |  |  | 1.000 |
| R0 | 70 | 50 |  | 30 | 30 |  |
| R1/R2 | 0 | 1 |  | 0 | 0 |  |
| Time to liquid diet (days) |  |  | **0.008** |  |  | 0.072 |
| Mean ± SD | 3.59±1.28 | 2.96±1.25 |  | 3.50±1.10 | 2.93±1.28 |  |
| Median (IQR) | 3.5 (3-4) | 3 (2-4) |  | 4 (3-4) | 2.5 (2-4) |  |
| Postoperative hospital stays (days) |  |  | **0.001** |  |  | **0.009** |
| Mean ± SD | 6.74±2.22 | 5.39±1.86 |  | 6.73±2.36 | 5.27±1.78 |  |
| Median (IQR) | 6 (5-8) | 5 (4-6) |  | 6 (5-8) | 5 (4-6) |  |
| Adverse events |  |  | **0.010** |  |  | 0.237 |
| Present | 1 | 7 |  | 0 | 3 |  |
| Absent | 69 | 44 |  | 30 | 27 |  |
| Imatinib treatment |  |  | 0.058 |  |  | 0.492 |
| Yes | 5 | 0 |  | 2 | 0 |  |
| No | 65 | 70 |  | 28 | 30 |  |
| Recurrence | 2 | 5 |  | 0 | 3 |  |

**Supplemental Table 7**

**Perioperative characteristics and long-term outcomes of LR and ER group in the entire cohort and after propensity score matching.**

Bold values indicate P<0.05.

HPF: High Power Field; SD: Standard Deviation; IQR: Interquartile Range; NIH: National Institutes of Health; LR: Laparoscopic resection; ER: Endoscopic resection.
