# Supplementary material for: Surgical Resection Is Still Better Than Endoscopic Resection for Patients With 2-5 cm Gastric Gastrointestinal Stromal Tumours: A Propensity Score Matching Analysis
Source: Front Oncol. 2021 Sep 15;11:737885. doi: 10.3389/fonc.2021.737885 (PMC8479163; doi:10.3389/fonc.2021.737885)
Supplement: Supplementary file 2 [file DataSheet_1.zip › Table_8.docx]

| Parameters | Entire cohort (before matching) | | *P*  value | Propensity score matched cohort | | *P*  value |
| --- | --- | --- | --- | --- | --- | --- |
|  | SR, n (%) | ER, n (%) |  | SR, n (%) | ER, n (%) |  |
| All cases | 159 | 6 |  | 12 | 6 |  |
| Age (years) |  |  | **0.039** |  |  | 1.000 |
| ≤ 60 | 70 | 0 |  | 0 | 0 |  |
| > 60 | 89 | 6 |  | 12 | 6 |  |
| Gender |  |  | 0.682 |  |  | 1.000 |
| Male | 79 | 2 |  | 4 | 2 |  |
| Female | 80 | 4 |  | 8 | 4 |  |
| BMI (kg/m^2^) |  |  | 0.296 |  |  | 1.000 |
| BMI < 18.5 | 6 | 1 |  | 2 | 1 |  |
| 18.5 ≤ BMI < 25 | 64 | 2 |  | 3 | 2 |  |
| BMI ≥ 25 | 89 | 3 |  | 7 | 3 |  |
| Comorbidities* |  |  | 0.432 |  |  | 1.000 |
| Present | 76 | 4 |  | 7 | 4 |  |
| Absent | 83 | 2 |  | 5 | 2 |  |
| Location |  |  | 0.506 |  |  | 1.000 |
| Fundus | 5 | 0 |  | 1 | 0 |  |
| Cardia | 77 | 5 |  | 8 | 5 |  |
| Body | 57 | 1 |  | 3 | 1 |  |
| Antrum | 20 | 0 |  | 0 | 0 |  |
| Tumor size |  |  | 0.207 |  |  | 0.976 |
| Mean ± SD | 4.31 ± 5.80 | 4.00 ± 5.48 |  | 4.00 ± 0.56 | 4.00 ± 5.48 |  |
| Median (IQR) | 4.5 (4.0-5.0) | 4.0 (3.5-4.25) |  | 4.0 (3.5-4.3) | 4.0 (3.5-4.25) |  |
| Mitotic index  (per 50 HPF) |  |  | 0.698 |  |  | 1.000 |
| 0-5 | 126 | 6 |  | 11 | 6 |  |
| 6-10 | 25 | 0 |  | 0 | 0 |  |
| >10 | 8 | 0 |  | 1 | 0 |  |
| Modified NIH risk |  |  | 0.568 |  |  | 0.676 |
| Very low | 4 | 0 |  | 2 | 0 |  |
| Low | 115 | 6 |  | 9 | 6 |  |
| Intermediate | 33 | 0 |  | 0 | 0 |  |
| High | 7 | 0 |  | 1 | 0 |  |
| Growth type |  |  | 1.000 |  |  | 1.000 |
| Intraluminal | 115 | 5 |  | 10 | 5 |  |
| Extraluminal | 44 | 1 |  | 2 | 1 |  |
| Shape |  |  | 0.369 |  |  | 0.333 |
| Regular | 148 | 5 |  | 12 | 5 |  |
| Irregular | 11 | 1 |  | 0 | 1 |  |
| Origin |  |  | 1.000 |  |  | 1.000 |
| Muscularis mucosal | 6 | 0 |  | 0 | 0 |  |
| Muscularis propria | 74 | 6 |  | 7 | 6 |  |
| NA | 80 | 0 |  | 5 | 0 |  |
| Ulceration |  |  | 1.000 |  |  | 1.000 |
| Present | 44 | 1 |  | 1 | 1 |  |
| Absent | 115 | 5 |  | 11 | 5 |  |
| High-risk imageology features† |  |  | **0.036** |  |  | 0.152 |
| Present | 99 | 1 |  | 7 | 1 |  |
| Absent | 60 | 5 |  | 5 | 5 |  |

**Supplemental Table 8**

**Baseline clinicopathologic characteristics of SR and ER group of 3-5 cm GISTs in the entire cohort and after propensity score matching.**

Bold values indicate P<0.05. BMI: Body Mass Index; SR: Surgical resection; ER: Endoscopic resection.

*Comorbidities: comprised of hypertension, diabetic mellitus, anemia, pulmonary disease (asthma, pneumonia, chronic obstructive pulmonary disease, etc.), heart disease (arrhythmia, coronary atherosclerotic heart disease, etc.), liver disease (hepatitis, cirrhosis, etc.), renal disease (nephritis, chronic kidney disease, etc.) and central nervous system disease (cerebrovascular disease, neurodegenerative disease, etc.).

†High-risk imageology features: heterogeneity, hyperechoic foci, or cystic spaces.
