# Supplementary material for: Surgical Resection Is Still Better Than Endoscopic Resection for Patients With 2-5 cm Gastric Gastrointestinal Stromal Tumours: A Propensity Score Matching Analysis
Source: Front Oncol. 2021 Sep 15;11:737885. doi: 10.3389/fonc.2021.737885 (PMC8479163; doi:10.3389/fonc.2021.737885)
Supplement: Supplementary file 2 [file DataSheet_1.zip › Table_9.docx]

| Parameters | Entire cohort (before matching) | | *P*  value | Propensity score matched cohort | | *P*  value |
| --- | --- | --- | --- | --- | --- | --- |
|  | SR, n (%) | ER, n (%) |  | SR, n (%) | ER, n (%) |  |
| All cases | 159 | 6 |  | 12 | 6 |  |
| Operate time (min) |  |  | 0.070 |  |  | **0.011** |
| Mean ± SD | 105.1 ± 42.2 | 73.3 ± 28.0 |  | 103.3 ± 107 | 73.3 ± 28.0 |  |
| Median (IQR) | 95 (75-130) | 65 (52.5-97.5) |  | 97.5 (91-120) | 65 (52.5-97.5) |  |
| En bloc resection |  |  | **<0.001** |  |  | **0.005** |
| Yes | 159 | 2 |  | 12 | 2 |  |
| No | 0 | 4 |  | 0 | 4 |  |
| Estimated blood loss (ml) |  |  | 0.339 |  |  | 0.529 |
| ≤ 50 | 129 | 6 |  | 10 | 6 |  |
| > 50 | 40 | 0 |  | 2 | 0 |  |
| Resection margin |  |  | 1.000 |  |  | 1.000 |
| R0 | 159 | 6 |  | 12 | 6 |  |
| R1/R2 | 0 | 0 |  | 0 | 0 |  |
| Time to liquid diet (days) |  |  | 0.381 |  |  | 0.523 |
| Mean ± SD | 3.47 ± 1.30 | 3.00 ± 0.89 |  | 3.33 ± 1.07 | 3.00 ± 0.89 |  |
| Median (IQR) | 3 (2-4) | 3 (2-4) |  | 4 (2-4) | 3 (2-4) |  |
| Postoperative hospital stays (days) |  |  | 0.091 |  |  | 0.301 |
| Mean ± SD | 7.04 ± 2.69 | 5.17 ± 0.75 |  | 6.00 ± 1.81 | 5.17 ± 0.75 |  |
| Median (IQR) | 6 (5-8) | 5 (4.75-6) |  | 5.5 (5-7.5) | 5 (4.75-6) |  |
| Adverse events |  |  | 0.202 |  |  | 1.000 |
| Present | 5 | 1 |  | 1 | 1 |  |
| Absent | 154 | 5 |  | 12 | 5 |  |
| Imatinib treatment |  |  | 1.000 |  |  | 1.000 |
| Yes | 22 | 0 |  | 1 | 0 |  |
| No | 137 | 6 |  | 11 | 6 |  |
| Recurrence | 11 | 1 |  | 0 | 1 |  |

**Supplemental Table 9**

**Perioperative characteristics and long-term outcomes of SR and ER group of 3-5 cm GISTs in the entire cohort and after propensity score matching.**

Bold values indicate P<0.05.

HPF: High Power Field; SD: Standard Deviation; IQR: Interquartile Range; NIH: National Institutes of Health; SR: Surgical resection; ER: Endoscopic resection.
